# Effect of Prestrain on the Actuation Characteristics of Dielectric Elastomers

**DOI:** 10.3390/polym12112694

**Published:** 2020-11-16

**Authors:** Mayank Kumar, Anutsek Sharma, Sakrit Hait, Sven Wießner, Gert Heinrich, Injamamul Arief, Kinsuk Naskar, Klaus Werner Stöckelhuber, Amit Das

**Affiliations:** 1 Leibniz-Institut für Polymerforschung Dresden e.V., 01069 Dresden, Germany; kumar.mayank96@gmail.com (M.K.); hait@ipfdd.de (S.H.); wiessner@ipfdd.de (S.W.); gheinrich@ipfdd.de (G.H.); 2 Rubber Technology Centre, Indian Institute of Technology, Kharagpur 721302, West Bengal, India; knaskar@rtc.iitkgp.ac.in; 3 Christian-Albrechts-Universität zu Kiel, 24143 Kiel, Germany; anutseks@gmail.com; 4 Institut für Werkstoffwissenschaft, Technische Universität Dresden, 01069 Dresden, Germany; 5 Institut für Textilmaschinen und Textile Hochleistungswerkstofftechnik, Technische Universität Dresden, 01069 Dresden, Germany; 6 Ingénierie des Matériaux Polymères, Université Claude Bernard Lyon1, 69100 Villeurbanne, France; arif.inji.chem1986@gmail.com; 7 Engineering and Natural Sciences, Tampere University, 33720 Tampere, Finland

**Keywords:** dielectric elastomers (DEs), nitrile rubber, hydrogenated nitrile rubber, actuation, stress relaxation, prestrain

## Abstract

Dielectric elastomers (DEs) represent a class of electroactive polymers that deform due to electrostatic attraction between oppositely charged electrodes under a varying electric field. Over the last couple of decades, DEs have garnered considerable attention due to their much-coveted actuation properties. As far as the precise measurement systems are concerned, however, there is no standard instrument or interface to quantify various related parameters, e.g., actuation stress, strain, voltage and creeping etc. In this communication, we present an in-depth study of dielectric actuation behavior of dielectric rubbers by the state-of-the-art *“Dresden Smart Rubber Analyzer” (DSRA)*, designed and developed in-house. The instrument allowed us to elucidate various factors that could influence the output efficiency of the DEs. Herein, several non-conventional DEs such as hydrogenated nitrile rubber, nitrile rubber with different acrylonitrile contents, were employed as an electro-active matrix. The effect of viscoelastic creeping on the prestrain, molecular architecture of the matrices, e.g., nitrile content of nitrile-butadiene rubber (NBR) etc., are also discussed in detail.

## 1. Introduction

Electroactive polymers (EAPs) are primarily a subset of soft active materials that offer actuation under electric fields [1,2]. The EAPs can further be categorized into electronic and ionic EAPs [3]. Dielectric elastomers (DEs) are basically electronic EAPs which offer obvious advantages over relatively ‘slow-response’ ionic counterparts [4]. Active materials derived from DEs are of great value in soft robotics and the development of stretchable electrodes [3,5], owing to their fast responses and considerably high electromechanical efficiency. Furthermore, dielectric elastomer actuators (DEA) offer large strain potentials, rendering conventional metal or semiconductor strain gauges unattractive. Following the rapid development in robotics and energy sectors, a surge in research on dielectric elastomer actuation has been reported in recent decades. It all apparently started with the work of Pelrine et al. [6] reporting high electromechanical transduction efficiency and huge strain, whereby DEAs found themselves at the center of interest. Furthermore, it has been shown that DEAs possess characteristic features closely resembling to that of biological muscles and thus, potentially offer bio-inspired solutions. To date, studies are concentrated mostly on the artificial muscles, soft robotics, flexible tactile display, and stretchable electrodes that are indeed a boon for future generations [7,8,9,10]. Therefore, for the applications described, DEs offer wide ranges of scope for research and development.

**Figure 1:**
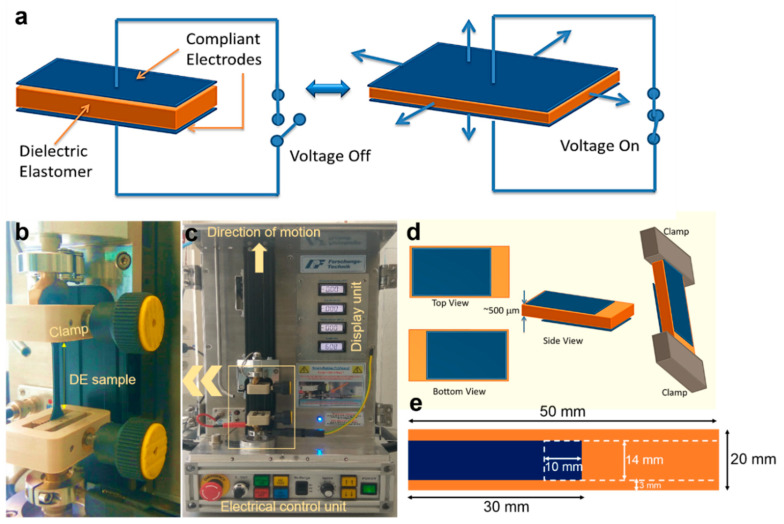
(**a**) Schematic representation of dielectric elastomer (DE) actuation upon application of an electrostatic force during voltage off and on state, respectively; (**b**,**c**) photographs of the Dresden Smart Rubber Analyzer (DRSA) with different units labeled. (**d**) Views of the fabricated electrodes from different viewing angle, and (**e**) dimension of the designed DE specimen (bottom).

The DEAs consist of a thin dielectric elastomer film sandwiched between two compliant electrodes on both sides, as shown in Figure 1. When an electric field is applied across the compliant electrodes, the electrostatic attraction between the opposite charges of both electrodes generates an electrostatic stress on the elastomeric film, causing it to contract in thickness and expand in area [11,12].

The fundamental parameter for characterizing dielectric actuators is the electrostatic pressure, also known as Maxwell stress (*σ*), which is generated when voltage is applied between the electrodes. It is defined as the change in electrostatic energy (*U*), per unit area *A*, per unit displacement of the film in the thickness direction *z* (the negative sign indicates that the generated stress is compressive):

(1) $$\sigma \;=\left(\frac{1}{A}\right)\left(-\frac{dU}{dz}\right)$$

The dielectric medium reduces the electrostatic force as compared to free space by a factor of $\varepsilon^{\prime }$ known as dielectric constant or relative permittivity. Considering elastomers as incompressible, any decrease in thickness results in an increase in the planar area. The electrostatic pressure exerted on a DE film subjected to an electric field is expressed as follows:

(2) $$\sigma =\varepsilon_{o}\varepsilon^{\prime }{\left(\frac{V}{z}\right)}^{2}=\;\varepsilon_{o}\varepsilon^{\prime }E^{2}$$

In the linear stress-strain regime, thickness strain (*S_z_*) is expressed in terms of Young’s modulus (Y),

(3) $$s_{z}=\;-\frac{\sigma }{Y}$$

where *z* = *z*_0_(1 + *s_z_*) and *z_0_* is initial thickness of the elastomer. Assuming incompressible nature of the matrix, we conveniently arrive at (1 + *S_z_*)(1 + *S_x_*)(1 + *S_y_*) = 1 and *S_x_* = *S_y_*. When compressive strain is very low, *z* can be replaced by *z_0_*

(4) $$s_{z}=-\frac{\varepsilon^{\prime }\varepsilon_{0}}{Y}{\left(\frac{V}{Z_{0}}\right)}^{2}$$

and in-plane strain *S_x_* = −0.5 *S_z_*, therefore,

(5) $$Sx=\frac{\varepsilon^{\prime }\varepsilon_{0}}{2Y}{\left(\frac{V}{Z_{0}}\right)}_{\;}$$

At low strain, the elastic strain energy density (*u_e_*) of DEA is expressed as,

(6) $$u_{e}==\frac{1}{2}\sigma S_{z}=-\frac{1}{2}\sigma S_{z}^{2}$$

Now if we consider z is not equal to *z_0_*, then from Equations (2) and (3) we can write:

(7) $$s_{z}=-\frac{\varepsilon^{\prime }\varepsilon_{0}}{Y}{\left\{\frac{V}{Z_{0}\left(1+z_{s}\right)}\right\}}^{2}$$

Following simplification,

(8) $$S_{z}^{3}+2S_{z}^{2}+S_{z}=-\left(\frac{\varepsilon^{\prime }\varepsilon_{0}}{Y}\right){\left(\frac{V}{Z_{0}}\right)}^{2}=b$$

where *b* is a parameter containing *V*. For a small increment of *V*, the change in the thickness strain (*S_z_*) with respect to *b* is given by the following differential,

(9) $$\frac{dS_{z}}{db}=\frac{1}{\left\{3S_{z}^{2}+4S_{z+}1\right\}}$$

According to Equation (4), actuation performance is directly related by the stiffness (Young’s modulus) and dielectric constant of the materials. Hence, they are expected to possess high dielectric strength and low dielectric losses, to maximize the stored energy and efficiency, thus avoid premature failures [13]. It is evident that the prime requirement of DEAs would also be able to withstand large voltages so that the actuation can as well be as large as possible. Additionally, DEAs are also expected to demonstrate strong compliance, i.e., the ability of stretching without breaking, and while remaining conductive. As exposed above, application of a voltage between the electrodes of a DEA can lead to large area expansion of the device. Its electrodes must be able to sustain the same strain without damage. In light of the above discussion, the parameters taken into considerations for the actuation of the material are: *(a)* relative permittivity of the material $(\varepsilon^{\prime }$), *(b)* change in film thickness and area (*z* and *A*), and *(c)* amount of voltage applied (*V*).

So far, a bulk of the studies conducted provide the actuations mostly in term of percentage, i.e., 10%, 40%, 100%, 200%; however, for specific applications like in soft robotics or even in artificial muscles and other biomimetic devices, each component requires a specific amount of actuating force [5,14]. Therefore, we propose the actuation in terms of relative force which has also been regarded as an ultimate motivation of the present study. 

Moreover, in addition to conventional silicone and acrylic rubber, researchers have developed different elastomers that generate significant actuation. Therefore, in this work, we chose acrylonitrile butadiene rubber (NBR) and hydrogenated-acrylonitrile butadiene rubber (HNBR)-based DEs due to their cost effectiveness, ready availability, and high permittivity [15]. The effect of a known dielectric filler (barium titanate, BaTiO_3_) onto the actuating performance of DEAs has also been investigated in this paper. 

## 2. Materials and Methods 

To analyze the actuation behavior at high strain and high voltage in order to obtain the actuation in terms of relative force, we have employed the state-of-the-art Dresden Smart Rubber Analyzer (DSRA) device. The DSRA was developed at Leibniz-Institut für Polymerforschung Dresden e.V. (IPF), Dresden, Germany. It is identical to a tensile machine; however, it uses a resistive sensor that records the resistance produced due to corresponding change in the geometry in the event of mechanical deformation (Figure 1b,c). The mechanical system was designed for tensile forces of up to 200 kN with the maximum pull out distance of 400 mm. An internally developed software Smart-Rubber.vxe together with the development tool Agilent VEE (Version 9.3, Agilent Technologies, Santa Clara, CA, USA) were used for this device. This enables the control of the linear drive of test machine and the high voltage unit, as well as the measurement of the force, pull-out distance, and the resistance at high voltage. A tensile test fixture with defined mechanical stress condition was created and any change in conductivity or/and resistance or, through application of controllable voltage, the change in force can be measured with time simultaneously. The major new modification in the device, as compared to another standard tensile test device, was observed with the integration of high voltage supply (of up to 10 kV) and the electrical contact. 

### 2.1. Material Used

It has been reported previously that mechanical and electrical properties of NBR and HNBR can be significantly modified by incorporating additives like cross-linkers, dielectric fillers, plasticizers and conductive filler [4,5]. In this work, we have employed a plasticizer and highly dielectric filler as additives to enhance the dielectric constant, and conductivity of composites. Typically, titanium dioxide (TiO_2_) and barium titanate (BaTiO_3_) have been widely used as a high dielectric filler to improve the dielectric constant of the synthetic rubber. For the sample preparation, a commercial grade NBR and HNBR (both from Arlanxeo, Dormagen, Germany) were used as high permittivity base elastomers. ZnO (Acros Organics, Schwerte, Germany) and Stearic acid (Fisher Chem Co., Schwerte, Germany) were employed as accelerator activator. As noted earlier, barium titanate (IV) (<100 nm particle size) procured from Sigma-Aldrich was used as dielectric nano-filler, having high permittivity ranging from 12,000 to 18,000. Dioctyl phthalate (DOP, obtained by Sigma-Aldrich, Munich, Germany) was utilized as plasticizer. CBS (N-cyclohexylbenzothiazylsulfenamide) and tetramethylthiuram disulfide (TMTD) were used as accelerators and sulfur as curing agents for NBR. Additionally, peroxide BIPB-40 (LUPEROX F 40, Safic Alcan, Bad Kreuznach, Germany) and coagent TAC (Rhenogran TAC-50, Rhein Chemie, Köln, Germany) were added to the HNBR matrix as curing agents. PRINTEX XE 2-B (conductive Carbon Black, Orion Engineered Carbons S.A., Frankfurt am Main, Germany) was mixed with few drops of the ammonium hydroxide (weak electrolyte, Acros Organics, Schwerte, Germany) for preparing an electrically conductive paste for fabrication onto the substrate. Different compositions of NBR and HNBR-based DE composites (in phr units) are shown in Table 1 and Table 2, respectively.

The ingredients shown in Table 1 and Table 2 were mixed in an internal mixer (Haake Rheomix) and a sheet of rubber was prepared on a roll-to-roll mill. Afterwards, it was cured in a mold of 0.5 mm thickness at 160 °C for all grades of NBR and at 180 °C and for HNBR under 200 N, respectively. For the comparison, all the samples were tested at a 30% prestrain and the actuation behavior was recorded and compared.

### 2.2. Fabrication of the Specimen

Several research groups have proposed different types of material for DEAs. Notably, Anderson et al. used silicone or acrylic rubbers along with a stretchable conducting material to obtain the desired actuation [16]. Silicone was also employed as DE to measure the electromechanical response of the sample [17]. Carlescu et al. adopted a sophisticated non-contact measurement system to monitor the gap between probe tip and metal target [17]. The probe consisted of coils which sent eddy currents to the target film and the motion of probe was sophisticatedly monitored by a tribo-meter connected to it. Any variation in the film thickness as a result of actuation due to applied voltage was recorded efficiently by this sophisticated probe. Aligned nylon fibers were also used to enhance the actuation of DEs; Shian et al. has worked on the development of inchworm robots with the help of an unimorph design [18]. In this respect, conducting rubber composites designed exclusively could be beneficial for fabrication of elastomeric dielectric actuators [19,20]. 

In the present work, we performed vulcanization followed by a rolled sheet cut into precise rectangular shapes with a typical specific dimension as shown in the Figure 1e. On each side, the electrode material was coated using a brush-coating technique. Figure 1d shows the top and the bottom view of the sample, respectively. Once the coating process was completed, the sample was then transferred to an oven for drying at 25 °C for 10 min. For uniform coating of electrodes, excess conductive carbon black was removed from the surface. The sample was then attached to the clamps in the DSRA (Figure 1a,d) sample holder and the program was set following the input entry of the prestrain (%), relaxation time (s), dimension of the sample (mm), strain load (mm/s) and voltage applied per unit time (kV/30 s). First, the sample was stretched under specified prestrain and then allowed to relax for 600 s. After the stress relaxation, voltage was applied subsequently every 30 s from 1 to 10 kV. It has been observed that prestraining the samples enhanced the dielectric breakdown strength [21,22]. This can be attributed to structural changes in the polymeric chain network that disrupt charge flow [21,22,23]. The process was repeated for different prestrains, i.e., 10%, 30%, 50%, 70% and 100%, and the actuation behavior was recorded with respect to force (N), time (s) and voltage (kV). The actuation characteristics of the NBR and HNBR substrates are discussed more precisely in the following section.

## 3. Results and Discussion

### 3.1. Actuation Behavior of Different Acrylonitrile Butadiene Rubber (NBR) Grades

For the fabrication of electrode, we have chosen NBR. Owing to the presence of polar -CN groups and high permittivity (ε = 18), NBRs are frequently employed as electrode substrates. Moreover, following the addition of dielectric filler and plasticizer, a further increment in the permittivity can be expected [17]. 

#### 3.1.1. At 30% Prestrain

To demonstrate the actuation profile of NBR, NBR 1846F was selected out of all the grades mentioned in Table 1. According to Equation (2), σ is inversely proportional to the thickness. Therefore, in order to obtain high actuation, 30% prestrain was applied [21]. 

**Figure 2:**
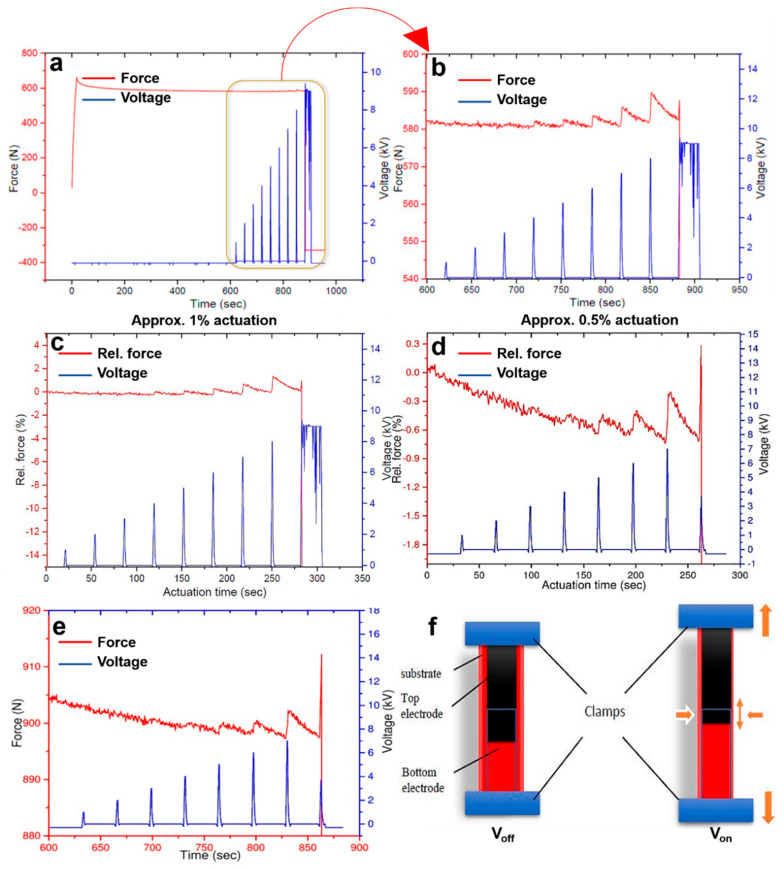
(**a**,**b**) Actuation profile of NBR 1846 F at 30% prestrain; (**c**) Actuation in terms of relative force (NBR with 30% prestrain) (**d**,**e**) Actuation profile of NBR 1846 F at 50% prestrain in terms of relative force (abbreviated as Rel. force, %) and force (N), respectively; (**f**) schematic illustration of actuation in voltage off and voltage on condition.

Figure 2a illustrates the complete actuation profile for NBR 1846F. The initial jump in force profile corresponds to the applied prestrain, followed by the stress relaxation for 600 s. Figure 2b shows the actuation peaks from 5 to 8 kV, whereas 9 kV is the breakdown voltage for the sample. The peaks appeared due to the compression in thickness and expansion in length of the actuator upon the application of prestrain. When the magnitude of voltage increases, expansion and compression of the actuator are shown to be increased as well. Therefore, peaks are increasingly prominent at higher voltages. As discussed earlier, we have expressed actuation in terms of relative force in this paper, to facilitate the applications of DEAs in the fields of soft robotics and artificial muscles. The estimated actuation in terms of relative force is shown in Figure 2c. As observed, approximately 1% actuation was reported for the 30% prestrained NBR sample. The distortion and subsequent actuation of the sample upon application of voltage are schematically explained in Figure 2f.

#### 3.1.2. At 50% Prestrain

To observe the change in actuation force, the experiment was also repeated at 50% prestrain. Figure 2d shows that maximum actuation of approximately 0.5% occurred at 7 kV. Figure 2e illustrates actuation peaks from 5 to 7 kV whereas, 8 kV is the breakdown voltage for the sample. For DEAs, a prestrain is often applied to thin the film and to increase the breakdown field. According to Equation (2), lowering the thickness would eventually elevate the actuation. However, it is not valid for every case. Nguyen et al. showed that the electrical resistance increases with the increase in prestrain, therefore, at 50% prestrain actuation is comparatively less than that at 30% prestrain [15]. Hence, for the rest of NBR grades, 30% prestrain was taken as an optimum for obtaining large actuation.

#### 3.1.3. Comparison of Actuation Characteristics with Increasing Acrylonitrile (ACN) Content

**Figure 3:**
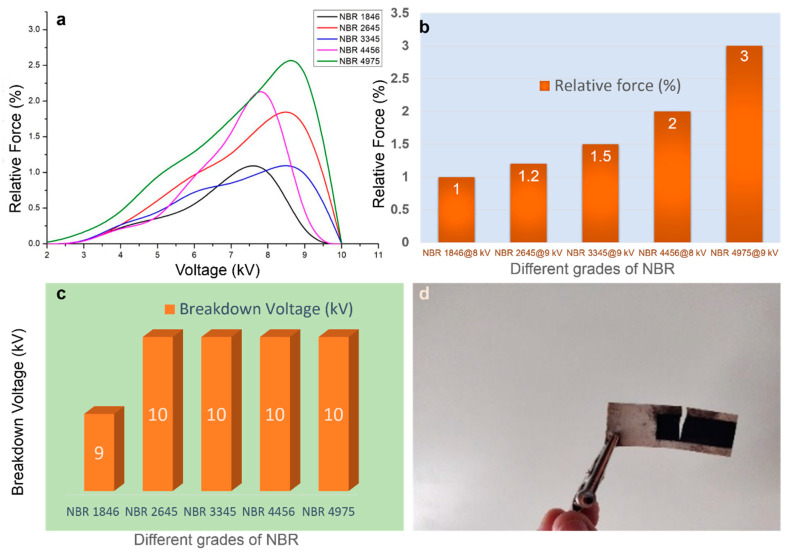
Effect of ACN content on actuation performance of NBR composites: (**a**) relative force (%) for various NBR grades as a function of voltage, (**b**) maximum actuation for different grades of NBR substrates; (**c**) breakdown voltages of different NBR grades, and (**d**) photograph of sample after testing.

Figure 3a,b compares the actuation performances of different grades of NBR substrates. It can also be deduced from the Figure 3a,b that, with increasing ACN content, a clear escalation in relative force of the actuator was observed. This hereby confirms that the polarity of the NBR substrate is explicitly associated and proportional to the actuation performance.

#### 3.1.4. Breakdown Voltage of NBR Grades

A dielectric substance or insulator normally becomes electrically charged when it is placed in an electric field. The substance is charged until a saturation point (until which it seems to be conducting electricity) is reached and upon saturation the dielectric substance or matrix no longer allows the flow of electricity, thus acting as an ideal insulator. Conversely, when a high electric field is applied across a dielectric substance, the electrons in the conduction band of atoms becomes excited and attain a higher kinetic energy, eventually resulting in conduction current. The conduction thus produced could be very high and can often result in melting, burning or vaporization of the dielectric substance. This process is known as the electrical breakdown of dielectric materials [24]. An exemplar of a sample that suffered electric breakdown in the current study can be viewed in Figure 3d.

The breakdown voltages of various NBR substrates are shown in Figure 3c. Except for NBR 1846 all other NBR samples exhibit almost similar value at 10 kV. The NBR 1846 comprised only 18 wt % nitrile content which is lower than all other NBR. This result implies that higher nitrile content may offer higher breakdown voltage of the crosslinked samples. 

### 3.2. Actuation Behavior of Hydrogenated-Acrylonitrile Butadiene Rubber (HNBR)

HNBR is hydrogenated NBR and has a saturated backbone. The HNBRs are known for aging resistance properties. Furthermore, due to the presence of different microstructure of the base polymer the dispersion of dielectric filler (barium titanate) and plasticizer (dioctyl phthalate, DOP) is more uniform as compared to the NBR matrix. As a result, HNBR possesses higher permittivity and consequently offers much higher actuation as compared to other elastomers [25]. Interaction of the polar groups present in HNBR could offer stronger interaction with fillers. Better dispersion of fillers in turn, leads to enhanced actuation performance. 

#### 3.2.1. Effect of Prestrain on Actuation

The actuation behavior of HNBR was examined at different prestrains (10%, 30%, 50%, 70%, 100%) in order to understand the effect of prestrain on actuator performance.

**Figure 4:**
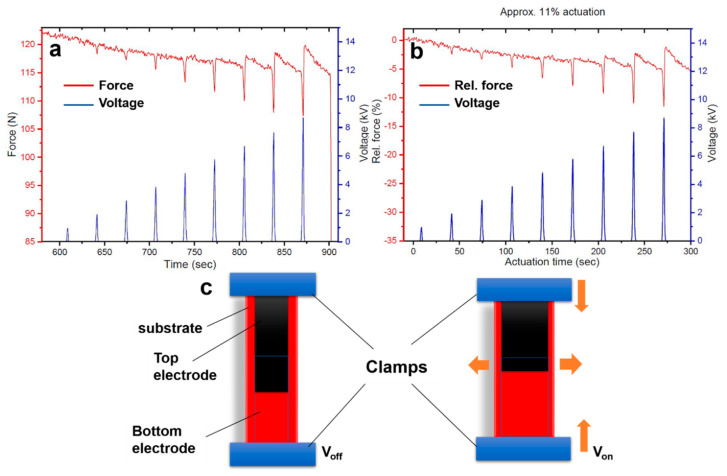
(**a**,**b**) actuation profile of HNBR substrate in terms of force and relative force (%), respectively, (**c**) schematic illustration of the mechanism of actuation for HNBR substrates.

Figure 4a,b demonstrates the actuation behavior of HNBR at 10% prestrain. The highest actuation of 11% is realized at 9 kV. Interestingly, the actuation force peaks are pointed downward, whereas that for NBR was upward. This can be attributed to a different (in contrast to NBR substrates in Figure 2f) actuation mechanism proposed in Figure 4c. For NBR samples, an increment in length and reduction in area were observed upon application of an electric field; however, for HNBR samples, shrinkage in length and expansion in total area was observed. Therefore, the actuation mechanism for HNBR is somehow different to that of the NBR. Most probably, the polymer chains under prestrain condition are oriented towards the stretching directions but when the voltage is applied, the dipole orientation of the polar segments of NBR and HNBR seems to be different. Further explanation cannot be provided in this paper and a more detailed study is required to unravel the exact mechanism behind this observation.

#### 3.2.2. Comparison of HNBR Actuation at Different Prestrains

**Figure 5:**
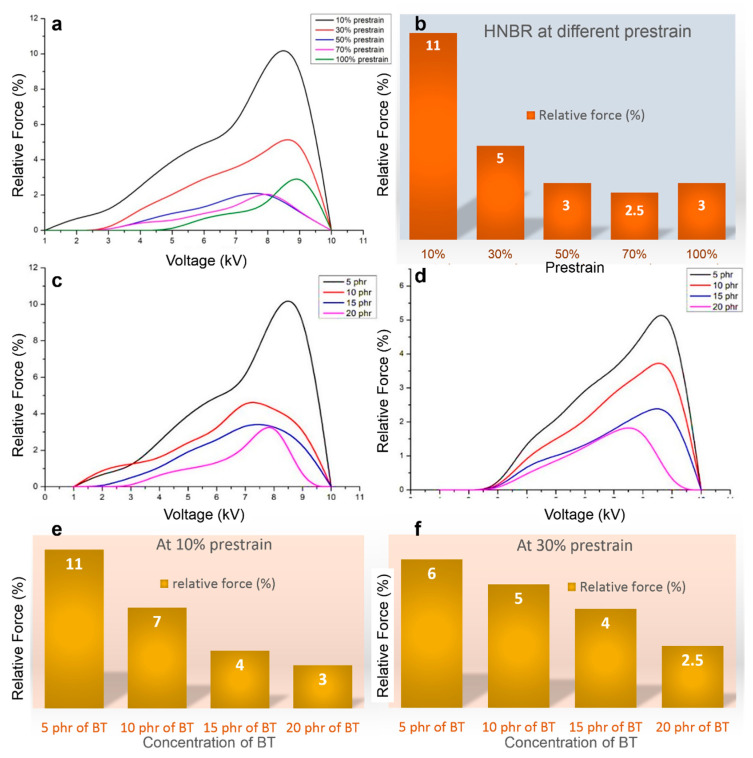
(**a**) Effect of prestrain on actuation performance of HNBR substrates; (**b**) maximum actuation of HNBR at different prestrains; effect of barium titanate (BT) concentration (phr) on the actuation performance (relative force (%) as function of voltage) at (**c**) 10% prestrain and (**d**) 30% prestrain; (**e**,**f**) the histogram representation of the actuation as a function of BT concentration at 10% and 30% prestrain, respectively.

Figure 5a shows the complete actuation profile of HNBR at different prestrains and voltages (1 to 10 kV). With an increase in applied voltage, a uniform increment in actuation was observed but near the breakdown voltage, the peaks fell rapidly. Also, a decrease in prestrain led to a progressive rise in the actuation performance (Figure 5b). In fact, 10% prestrain in HNBR substrate resulted in the highest observed actuation (11%), and as the prestrain was increased, the actuation was observed to fall systematically due to an increase in electrical resistance. However, beyond 70% prestrain the actuation was increased by 0.5% for 100% prestrain. Apparently, this interesting trend can be explained as follows: according to Maxwell stress, lowering the thickness gives rise to increased actuation. On the other hand, the higher the prestrain, the higher will be the electrical resistance. Therefore, after 70% prestrain the reason for the increase in actuation due to reduction in thickness (and hence higher actuation) dominates over the electrical resistance contribution, albeit to little extent. Hence, actuation rises by 0.5%.

#### 3.2.3. Comparison of HNBR Actuation with Varying Barium Titanate (BT) Contents at Different Prestrains

Actuation performances were also investigated at different dielectric filler (barium titanate, BaTiO_3_) content and are shown in Figure 5c,d. For comparison, two cases were considered, i.e., 10% prestrain and 30% prestrain. As expected, upon increase in barium titanate contents, a decrease in actuation behavior was observed for both prestrains. This finding echoes the results obtained from the histograms in Figure 5e,f. The histograms from Figure 5e,f illustrate that an increase in BT concentration deteriorates the actuation performance. This is explained in terms of poor dispersion of the BT nanofiller, resulting in agglomeration. Increased BT substitution actually enhances the elastic moduli of the HNBR composites, thus the gain in dielectric performances is actually overshadowed by the increased mechanical stiffness. Dispersion stability is also affected by lack of unsaturation in HNBR. The results further confirm that 5 phr of BT is optimal for the actuator and the optimal prestrain is 10% for HNBR.

## 4. Conclusions

We have incorporated NBR and HNBR substrate for electrode fabrication primarily due to their cost-effectiveness, easy processability, high permittivity and compatibility to various fillers and additives. Additionally, in order to further ameliorate the said properties, we have incorporated various fillers (dielectric inclusions, plasticizers). NBR-based electrodes provide descent actuation due to their high permittivity i.e., ε = 18 (more than any conventional rubber can offer) and the results showed that, with the gradual increment in ACN content in NBR (from 18% to 49%), the actuation actually rises from 1% to 3%. The second study involving HNBR demonstrated superior results owing to its high permittivity provided by the polar contents (i.e., H^+^ and CN^−^) present, as they can conveniently attach to polar dielectric filler (barium titanate) and plasticizer and can withstand up to 10 kV. At 10% prestrain, HNBR showed the highest actuation of 11% and as the prestrain is increased, actuation decreases. Moreover, 5 phr of BT with 10 phr of DOP is shown to be the optimum for maximum actuation. As the BT content is increased, dispersion becomes poor due to shear-induced agglomeration of nanofillers during mixing. 

## Figures and Tables

**Table 1:** Composition of various acrylonitrile butadiene rubber (NBR)-based DEs (in phr).

Ingredients ^1^	M1	M2	M3	M4	M5	M6	M7	M8	M9
NBR 1846 F	100	-	-	-	-	-	-	-	-
NBR 2645 F	-	100	-	-	-	-	-	-	-
NBR 3345 F	-	-	100	-	-	-	-	-	-
NBR 4456 F	-	-	-	100	-	-	-	-	-
NBR 4975 F	-	-	-	-	100				
HNBR	-	-	-	-	-	100	100	100	100
ZnO	5	5	5	5	5	5	5	5	5
Stearic acid	2	2	2	2	2	2	2	2	2
Barium titanate	5	5	5	5	5	5	10	15	20
Dioctyl phthalate	10	10	10	10	10	10	10	10	10
CBS	1.5	1.5	1.5	1.5	1.5	-	-	-	-
TMTD	1	1	1	1	1	-	-	-	-
Sulfur	2	2	2	2	2	-	-	-	-
BIBP 40	-	-	-	-	-	2	2	2	2
TAC	-	-	-	-	-	1	1	1	1

^1^ phr (parts per hundred rubber).

**Table 2:** Composition of hydrogenated-acrylonitrile butadiene rubber (HNBR) composites with different filler contents (in phr).

Ingredients ^1^	1	2	3	4
HNBR C 3446	100	100	100	100
ZnO	5	5	5	5
Stearic acid	2	2	2	2
Barium titanate	5	10	15	20
DOP	10	10	10	10
BIPB 40	2	2	2	2
TAC	1	1	1	1

^1^ in phr (parts per hundred rubber).
